# Engrailed-1 in eccrine sweat gland development and regeneration: from evolutionary innovation to therapeutic perspectives

**DOI:** 10.3389/fcell.2026.1917336

**Published:** 2026-10-01

**Authors:** Lei Zhang, Zizhen Zhang, Jiaqi Cheng, Junhong Zhao, Yanyi Xing, Xiang Liu, Lihong Peng, Sixun Wu, Haihong Li

**Affiliations:** 1 Department of Psychiatry and Clinical Psychology, Seventh Affiliated Hospital, Sun Yat-sen University, Shenzhen, Guangdong, China; 2 The First School of Clinical Medicine, Hubei University of Medicine, Shiyan, Hubei, China; 3 Department of Burns and Plastic Surgery, The Second Affiliated Hospital, Shantou University Medical College, Shantou, Guangdong, China; 4 Department of Wound Repair and Dermatologic Surgery, Taihe Hospital, Hubei University of Medicine, Shiyan, Hubei, China; 5 Department of Burns and Plastic Surgery, Seventh Affiliated Hospital, Sun Yat-sen University, Shenzhen, Guangdong, China

**Keywords:** cell fate specification, developmental hierarchy, eccrine sweat glands, Engrailed‐1 (EN1), regenerative Medicine

## Abstract

Functional regeneration of eccrine sweat glands (ESGs) remains a major unmet need in burns and trauma, where extensive skin loss leads to permanent anhidrosis, life-threatening hyperthermia, and impaired quality of life. Here we synthesize evidence pointing to the homeodomain transcription factor Engrailed-1 (EN1) as a central regulator that coordinates ESG development across four interconnected biological functions: (1) establishing a permissive ventral limb field by repressing the WNT7A–LMX1B dorsalization pathway; (2) executing a binary fate switch that suppresses the hair follicle program while activating a pro-glandular program; (3) functioning as a quantitative rheostat whose expression levels determine ESG density—a mechanism potentiated by primate-specific enhancers such as ECE18; and (4) being required for the establishment of a transient eccrine niche (EDEN) that provides mesenchymal support during gland morphogenesis in mouse models. Drawing on this framework, we propose a tiered pathogenesis–regeneration model (Tiers 1–4) mapping to distinct EN1 failures: Tier 1 (ventral field loss) points to bioengineered glabrous skin substitutes; Tier 2 (lineage specification failure) guides strategies to redirect progenitor fate; Tier 3 (aborted morphogenesis) highlights the need for EDEN-like niche reconstruction; and Tier 4 (compromised adult integrity) suggests approaches to reinforce the mechanical anchor of existing glands through EN1-mediated regulation of epidermal isoform of Bullous Pemphigoid Antigen 1 (BPAG1e), a proposed function inferred from *in vitro* evidence but untested *in vivo*. This multi-tiered model offers a conceptual framework for functional ESG restoration while addressing translational gaps between embryonic programs and adult tissue competency—a framework that remains speculative and requires rigorous validation in human tissues, as many mechanisms are supported primarily by mouse studies.

## 1 Introduction

The eccrine sweat gland (ESG) is a cornerstone of human evolutionary success, constituting the primary organ of thermoregulation that enabled endurance running and the colonization of diverse climates by the genus *Homo* (Best and Kamilar, 2018; Best et al., 2019; Folk and Semken, 1991). Unlike most mammals, in which ESGs are restricted to friction pads, humans possess an extraordinary density of 2–4 million functional ESGs across the entire body surface—a derived trait critical for evaporative cooling (Cui and Schlessinger, 2015; Kamberov et al., 2015; Saga, 2002). Clinically, ESG dysfunction—whether due to congenital disorders such as hypohidrotic ectodermal dysplasia (HED), severe burns or trauma—leads to life-threatening hyperthermia and significantly diminished quality of life (Cui and Schlessinger, 2015; Gaide and Schneider, 2003). Despite this clinical urgency, ESG regeneration has been fundamentally hindered by an incomplete understanding of the key genetic regulators that orchestrate the entire developmental continuum from skin field establishment to terminal differentiation (Lin et al., 2021; Pontiggia, 2017).

The discovery of the homeodomain transcription factor Engrailed-1 (EN1) as a key controller has begun to fill this gap, but a comprehensive understanding of its full regulatory scope—from embryonic patterning to adult homeostasis—has only emerged through studies over the past decade (Danielian and McMahon, 1996; Loomis et al., 1996; Lu et al., 2016). The story of *EN1* is one of deep evolutionary conservation and functional repurposing. Initially characterized for its role in segmental patterning in *Drosophila* and later for its indispensable function in establishing the dorsal-ventral (DV) axis of the vertebrate limb, EN1 has been recurrently co-opted for novel functions throughout evolution (Gibert, 2002; Ma et al., 2024). During limb development, *EN1* is expressed in the ventral ectoderm, where it represses the dorsal determinant *WNT7A*, thereby specifying ventral identity and creating the glabrous (hairless) skin of the palms and soles (Loomis et al., 1996; Tsugane and Yasuda, 1998). This establishes the anatomical and molecular preconditions that are permissive for subsequent ESG development. The catastrophic consequence of losing this repression is revealed in *En1* null mice, where ectopic *Wnt7a* expression results in a “double dorsal” limb, complete with loss of footpads and transformation of ventral skin into hair-bearing, dorsal-type epidermis (Loomis et al., 1996; Wurst et al., 1994).

Within this established glabrous field, EN1 performs a second, decisive function: it acts as a binary genetic switch for ESG lineage specification. Integrating upstream signals from Bone Morphogenetic Protein (BMP) and Fibroblast Growth Factor (FGF) pathways, EN1 acts as a key downstream mediator in epidermal progenitors to suppress the hair follicle (HF) program (in part by enabling the repression of Sonic Hedgehog (SHH) signaling) while simultaneously activating a pro-glandular transcriptional program (Lu et al., 2016). Notably, the level of EN1 expression functions as a quantitative rheostat, determining local ESG density (Kamberov et al., 2015). This dosage-sensitive mechanism was dramatically leveraged during human evolution: the emergence of the human-specific enhancer *EN1* candidate enhancer 18 (*hECE18*) drove elevated epidermal *EN1* expression, contributing to our species’ exceptional sweat gland density and thermoregulatory capacity (Aldea et al., 2021; Aldea and Kamberov, 2022). Beyond fate specification, EN1 further orchestrates gland morphogenesis by inducing a specialized, transient dermal microenvironment—the EN1-dependent eccrine niche (EDEN)—which provides essential signaling and structural support in mouse models (Branch and Ezhkova, 2024; Dingwall et al., 2024). Postnatally, EN1 has been proposed to maintain adult gland integrity through transcriptional regulation of structural components like the epidermal isoform of Bullous Pemphigoid Antigen 1 (BPAG1e) —a regulatory relationship established *in vitro* for the related Engrailed proteins and inferred for EN1 based on its sustained expression in adult glands—although this adult maintenance function remains to be directly tested *in vivo* (Mainguy et al., 1999; Mainguy et al., 2000).

In this review, we synthesize the evidence for this paradigm across evolutionary, developmental, and homeostatic contexts. We then propose a stage-specific therapeutic framework for functional ESG regeneration based on a tiered pathogenesis–regeneration model serving as a conceptual framework. Throughout, we explicitly distinguish between well-established mechanisms and working hypotheses, and we critically examine the remaining gaps in our understanding—including the limitations of mouse models, the incomplete characterization of human EN1 targets, the unresolved molecular cue for EDEN induction, and the challenges of translating developmental mechanisms into adult regeneration. We also acknowledge that several components of this framework, particularly the EDEN induction model and the proposed ductal collapse mechanism in Tier 4, remain speculative and await direct experimental validation in human systems.

## 2 EN1: Evolutionary origins and molecular architecture

### 2.1 Deep evolutionary conservation and vertebrate diversification

The evolutionary trajectory of *EN1* traces back to a single ancestral *engrailed* gene in the last common ancestor of bilaterians (De Robertis, 2008; Gibert, 2002). This primordial gene played fundamental roles in neurogenesis and, through intercalary evolution, was later recruited for segmentation in lineages like arthropods, where *engrailed* is essential for establishing segment polarity (Martinez Arias et al., 1988; Patel et al., 1989). The two rounds of whole-genome duplication (WGD) in early vertebrates provided the genetic substrate for diversification (Dehal and Boore, 2005). The ancestral gene duplicated, giving rise to *En1* and *Engrailed-2* (*En2*) (Joyner and Martin, 1987). Following duplication, *En1* and *En2* underwent subfunctionalization, partitioning ancestral roles while each acquired novel functions (Hanks et al., 1995). *EN1* retained ancestral roles in patterning the nervous system (e.g., midbrain-hindbrain boundary organizer) but was co-opted for a novel role in vertebrate limb development—a dramatic functional shift from anterior-posterior (AP) to dorsal-ventral (DV) axis patterning (Loomis et al., 1996; Wurst et al., 1994). This specialization is so profound that *Drosophila engrailed* can partially rescue mouse *En1* mutants in the brain but not in the limb, underscoring the unique, acquired function of *EN1* in DV patterning (Hanks et al., 1998). Beyond development, *EN1* has acquired essential roles in adult neuroprotection, where its loss leads to progressive degeneration of dopaminergic neurons, recapitulating features of Parkinson’s disease (Alvarez-Fischer et al., 2011; Rekaik et al., 2015).

### 2.2 Molecular architecture: a toolkit for transcriptional and paracrine signaling

The functional versatility of EN1 is reflected in its protein structure, which supports its roles as a transcription factor and, potentially, as a secreted paracrine signaling molecule (Joliot et al., 1998; Morgan, 2006) (Figure 1; summarized in Table 1). Understanding this duality is essential for grasping how EN1 can orchestrate both cell-autonomous fate decisions and non-cell-autonomous niche induction.

**FIGURE 1:**
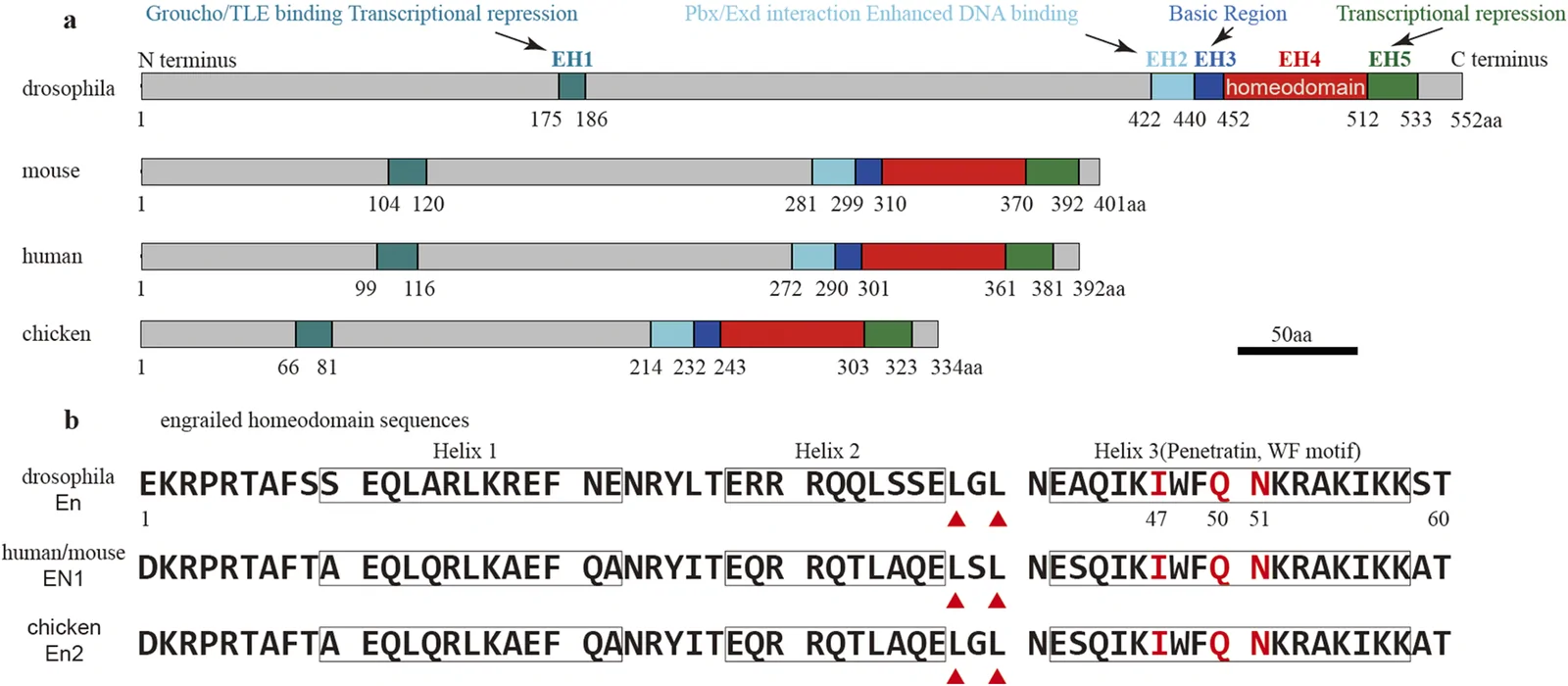
Modular domain architecture of EN1 homeoprotein. (Source: The figures are structural schematic based on published sequence alignments; Created with DNAMAN). **(a)** Domain structure of EN in *Drosophila*, and EN1 in mouse, human, and chicken. Five conserved EH domains (EH1-EH5) mediate repression and cofactor interactions (Logan et al., 1992). Amino acid numbering and a 50-amino acid (aa) scale bar are shown. **(b)** Homeodomain (EH4) sequence alignment of *Drosophila* EN, human EN1 (highly conserved between human and mouse), and chicken EN2 (Ades and Sauer, 1995; Bürglin and Affolter, 2016; Logan et al., 1992). Helices 1-3 are boxed. Residues 47, 50, and 51 (shown in red) are located in the recognition helix (helix 3) and are responsible for base-specific contacts in the DNA major groove. Arrowheads indicate conserved leucine residues between helices 2 and 3, which form part of the nuclear export signal (NES). Sequence sources: *Drosophila* EN (UniProt P02836), mouse EN1 (UniProt P09065), human EN1 (UniProt Q05925), chicken EN1 (UniProt Q05916), chicken EN2 (UniProt Q05917). Domain assignments are based on published sequence alignments (Logan et al., 1992), and the structural schematics represent the authors’ interpretation of the published sequence features.

**TABLE 1:** Molecular architecture of EN1 and its regulatory network in eccrine sweat gland development.

Category	Component	Function/Role	Evidence level	Experimental evidence (key methods and key quantitative data)	Key references
Protein Domains	EH1	Core repression motif; recruits Groucho/TLE corepressors to mediate transcriptional silencing via HDAC recruitment	Definitive	Domain mapping; protein-protein interaction assays (*in vitro* and *in vivo*)	( Copley, 2005; Smith and Jaynes, 1996 )
	EH2	Mediates interaction with Pbx/Exd cofactors; enhances DNA-binding specificity; enables context-dependent switching between repression and activation	Definitive (*Drosophila*)/Correlative (mammals)	Yeast two-hybrid; EMSA	( Fujioka et al., 2012; Kobayashi et al., 2003 )
	EH3	Conserved domain; function remains unknown	Speculative (structural inference)	No experimental data available	(Logan et al., 1992)
	EH4 (Homeodomain)	60-aa helix-turn-helix DNA-binding domain; recognition helix mediates base-specific contacts in DNA major groove	Definitive	X-ray crystallography	( Bürglin and Affolter, 2016; Kissinger et al., 1990 )
	EH5	Contributes to transcriptional repression and paralog specificity	Definitive	Transcriptional repression assays	(Tolkunova et al., 1998)
	NES	Leucine-rich motif between helices 2–3; mediates CRM1-dependent nuclear export and targets EN1 to caveolae for unconventional secretion	Definitive	Mutagenesis; secretion assays; CK2 phosphorylation regulates nuclear retention	( Joliot et al., 1998; Maizel et al., 2002 )
	Penetratin motif	WF dipeptide in helix 3; mediates energy-independent membrane translocation	Definitive (*in vitro*)/Speculative (*in vivo*)	Cell uptake assays; *in vivo* functional significance remains untested	(Dom et al., 2003)
Upstream Regulators	DEAF1	Direct transcriptional activator; binds conserved upstream sites; *Deaf1* KO phenocopies partial *En1* LOF	Definitive (mouse)	ChIP; KO mouse: ↓*En1* expression, ↓ESG number	(Aldea et al., 2019)
	BMP/FGF signaling	Act upstream of EN1 in a genetic pathway; mesenchymal BMP/FGF signaling is required to create a permissive signaling environment for EN1 to execute its pro-glandular function (e.g., by suppressing SHH)	Definitive (mouse models)	Epithelial *Bmpr1a* KO converts footpad sweat glands into HF-like structures, establishing that BMP signaling acts upstream of EN1 in fate determination. Ectopic Bmp5, Bmp4, or Fgf18 in dorsal skin upregulates *En1* mRNA, but direct causality remains untested; correlative expression in ventral foot skin supports this link	(Lu et al., 2016)
	ECE18 enhancer (human)	Primate-specific enhancer; 12 lineage-specific mutations; SP1^A^ and SP1^B^ create high-affinity SP1 binding sites; linked to increased eccrine gland density	Definitive (human cells and transgenic mice)	Luciferase reporter: ∼13-fold↑ activity; CRISPRi: ↓∼40% *EN1*; mouse knock-in: ↑ESG density	(Aldea et al., 2021)
	ECE20 enhancer (conserved)	Conserved pleiotropic enhancer; required for *EN1* in skin and midbrain	Strong (mouse)	Mouse KO: ↓∼20% *En1*; ↓∼30% ESG number	(Aldea et al., 2023)
​	*MAENLI* lncRNA	Limb-specific *EN1* regulator; non-coding deletions cause severe limb patterning defects	Correlative (human genetics)	Human deletion mutation analysis	(Allou et al., 2021)
Downstream Targets (Activated)	*Dkk4*	Wnt feedback inhibitor; establishes glandular field; reinforces BMP-SHH antagonism	Correlative (mouse)	snRNA-seq identifies *Dkk4* as an En1-dependent, eccrine-enriched transcript; direct EN1 occupancy not demonstrated	( Dingwall et al., 2024; Lu et al., 2016 )
	*Bpag1e*	Hemidesmosome component; anchors cytoskeleton to basement membrane; proposed role in maintaining adult gland integrity	Correlative *(in vitro)*/Speculative *(in vivo)* |	EMSA and DNase I footprinting demonstrate Engrailed homeodomain (EnHD) binding to *Bpag1e* promoter *in vitro*; promoter-reporter assays in keratinocyte cell lines show context-dependent transcriptional regulation (repression by full-length En2, while an N-terminally truncated En2 acts as a dominant-negative and relieves this repression); adult gland maintenance function untested *in vivo*	( Mainguy et al., 1999; Mainguy et al., 2000 )
Downstream Targets (Repressed)	*Shh*	Canonical HF inducer; its suppression permits ESG fate	Speculative	Upregulated upon En1 loss, but direct repression by EN1 has not been established	(Lu et al., 2016)
Indirect Effects	FOXA1, BEST2, NKCC1	Terminal secretory differentiation markers; not direct EN1 targets	Definitive (mouse)	Mouse KO (*Foxa1*): anhidrosis, normal gland morphology; ↓*Best2*, ↓*Nkcc1*	(Cui et al., 2012)
Niche Induction (working model)	EDEN (*S100a4* ^*+*^ */Tnc* ^*+*^ fibroblasts)	Transient fibroblast population; provides mesenchymal support for gland morphogenesis	Strong (mouse)/Speculative (human)	Lineage tracing; S100a4^+^ cell ablation: ↓∼45% ESG formation; scRNA-seq confirms marker profile; EN1-derived cue remains speculative	(Dingwall et al., 2024)
Pathological Roles (Safety)	Oncogenic transformation	EN1 overexpression promotes cell survival in basal-like and triple-negative breast cancer; correlates with poor survival and brain metastases	Correlative (human studies)	Overexpression/knockdown; patient sample analysis; correlative survival data	( Beltran et al., 2014; Peluffo et al., 2019 )
	Pro-fibrotic effects	EN1 amplifies TGF-β signaling; promotes myofibroblast differentiation; EPFs drive scarring; mechanical tension via YAP activates EN1 in wound healing; EN1 silencing reverses profibrotic effects and enables scarless regeneration	Definitive (mouse and human fibroblasts)	Fibroblast lineage tracing; fibroblast-specific *En1* KO → ↓fibrosis; YAP inhibition or fibroblast-specific *Yap* KO → prevents EN1 activation → promotes wound regeneration; human SSc fibroblasts: EN1 re-expressed; EN1-targeted siRNA (FibroMC) → silences EN1 → prevents scarring	( Györfi et al., 2021; Mascharak et al., 2021; Rinkevich et al., 2015; Xu et al., 2025 )

Abbreviations: aa, amino acid; ChIP, chromatin immunoprecipitation; CRISPRi, CRISPR, interference; CRM1, Chromosome Region Maintenance 1; EMSA, electrophoretic mobility shift assay; ESG, eccrine sweat gland; FGF, fibroblast growth factor; HDAC, histone deacetylase; IHC, immunohistochemistry; KO, knockout; LOF, loss of function; NES, nuclear export signal; scRNA-seq, single-cell RNA, sequencing; SSc, systemic sclerosis; TLE, Transducin-like enhancer of split.

Evidence Level Definitions: Definitive = multiple lines of evidence (genetic + biochemical + functional) validated *in vivo*, with cross-species validation where available; Strong = primarily from a single model system (e.g., mouse), lacking cross-species validation; Correlative = expression or phenotypic association without functional validation; Speculative = theoretical inference, no direct experimental evidence.

#### 2.2.1 Structural determinants of transcriptional activity

The EN1 protein contains five highly conserved Engrailed Homology (EH) domains that collectively underpin its canonical transcription factor function (Logan et al., 1992) (Figure 1). The EH1 domain is a core repression motif that recruits Groucho/Transducin-like enhancer of split (TLE) corepressors, mediating transcriptional silencing through histone deacetylase recruitment (Copley, 2005; Smith and Jaynes, 1996) (Figure 1). The EH2 domain mediates interaction with Pbx/Exd cofactors, enhancing DNA-binding specificity and allowing context-dependent switching between repressive and activating functions—a mechanistic feature that may explain EN1’s ability to both repress *Shh* and activate glandular targets (Fujioka et al., 2012; Kobayashi et al., 2003) (Figure 1). The Homeodomain (EH4) is a 60-amino acid helix-turn-helix (HTH) domain responsible for DNA binding; the third “recognition helix” makes base-specific contacts in the DNA major groove via conserved residues at positions 47, 50, and 51 (Ades and Sauer, 1995; Bürglin and Affolter, 2016; Kissinger et al., 1990) (Figure 1). The EH5 domain contributes to transcriptional repression and paralog specificity (Tolkunova et al., 1998) (Figure 1).

#### 2.2.2 Motifs governing unconventional secretion and intercellular transfer

EN1 has been shown to be secreted and internalized via a non-canonical pathway that bypasses the ER-Golgi apparatus in cell culture systems. Joliot et al. demonstrated that EN1 expressed in COS cells can be recovered in abutting primary neurons, and that approximately 5% of EN1 is protected within membrane fractions against proteinase K proteolysis, suggesting access to a luminal compartment prerequisite for secretion. (Joliot et al., 1998). A leucine-rich Nuclear Export Signal (NES) between helices 2 and 3 mediates nuclear export via CRM1 and targets EN1 to caveolae for secretion, with secretion regulated by casein kinase 2 (CK2) phosphorylation (Maizel et al., 2002) (Figure 1). The third alpha-helix contains the Penetratin motif with a conserved tryptophan-phenylalanine (WF) dipeptide that mediates membrane translocation (Dom et al., 2003; Joliot et al., 1998) (Figure 1). Through this machinery, EN1 has been proposed to function as a paracrine signaling molecule—a role supported by its neurotrophic activity on spinal cord α-motoneurons and by paracrine functions demonstrated for the related homeoprotein EN2. However, it is important to emphasize that while the secretion machinery has been rigorously characterized in cell culture, its functional significance *in vivo*—particularly for EDEN induction during ESG development—remains speculative, as no conditional alleles are currently available to separate EN1’s transcriptional from its paracrine functions (see Section 3.4; Table 1).

### 2.3 Cis-regulatory evolution as the driver of functional diversification

The remarkable functional diversification of *EN1* was driven primarily by changes in its *cis*-regulatory elements (CREs), rather than its protein-coding sequence, which is highly conserved across vertebrates (Gibert, 2002). The modular organization of the *EN1* regulatory landscape, comprising pleiotropic enhancers active in multiple tissues and tissue-specific fine-tuners, provides a paradigm for how developmental regulators can evolve new functions without compromising essential ancestral roles. For example, the limb-specific expression of *EN1* is controlled by a complex regulatory hub involving the *Maenli* lncRNA and temporal enhancers LSEE1/2, deletions of which cause severe dorsal-ventral patterning defects (Allou et al., 2021; Ringel et al., 2026). This principle of CRE-driven innovation is central to understanding how the primate-specific ECE18 enhancer later evolved to boost *EN1* expression in sweat gland precursors, a key event in human thermoregulatory evolution (as detailed in Section 2.2).

### 2.4 Evolution of eccrine sweat glands and the role of EN1

The ESG is a pivotal adaptive innovation in mammalian evolution, particularly within the primate lineage, where it transitioned from a tactile organ to a sophisticated thermoregulatory system (Best and Kamilar, 2018). The central role of *EN1* in this trajectory is as a quantitative determinant of appendage fate, a mechanism that appears to be conserved across mammals but whose regulatory inputs have been profoundly modified during primate evolution.

### 2.5 Phylogenetic distribution of ESGs

The evolutionary origins of ESGs trace back to synapsid reptiles over 200 million years ago, with true ESGs emerging by the Paleocene epoch (Folk and Semken, 1991). In extant non-primate mammals (e.g., rats and mice), strepsirrhine primates (e.g., lemurs and lorises), and most New World monkeys (e.g., silvered sakiwinki and the owl monkey), ESGs are strictly confined to glabrous skin on friction pads on palmar and plantar surfaces (Figure 2). These regions endure high mechanical stress and serve primarily to enhance grip stability and tactile sensitivity, adaptations essential for arboreal locomotion and precision foraging. An additional specialization occurs in certain New World monkeys with prehensile tails (e.g., woolly, spider, and black howler monkeys), where ESGs also appear on ventral tail skin, further improving grasp and dexterity during brachiation (Figure 2). Collectively, these patterns underscore that in most mammalian lineages, ESGs evolved primarily for non-thermoregulatory, mechanosensory roles (Best and Kamilar, 2018; Ellis and Montagna, 1962; Machida and Perkins, 1966) (Figure 2).

**FIGURE 2:**
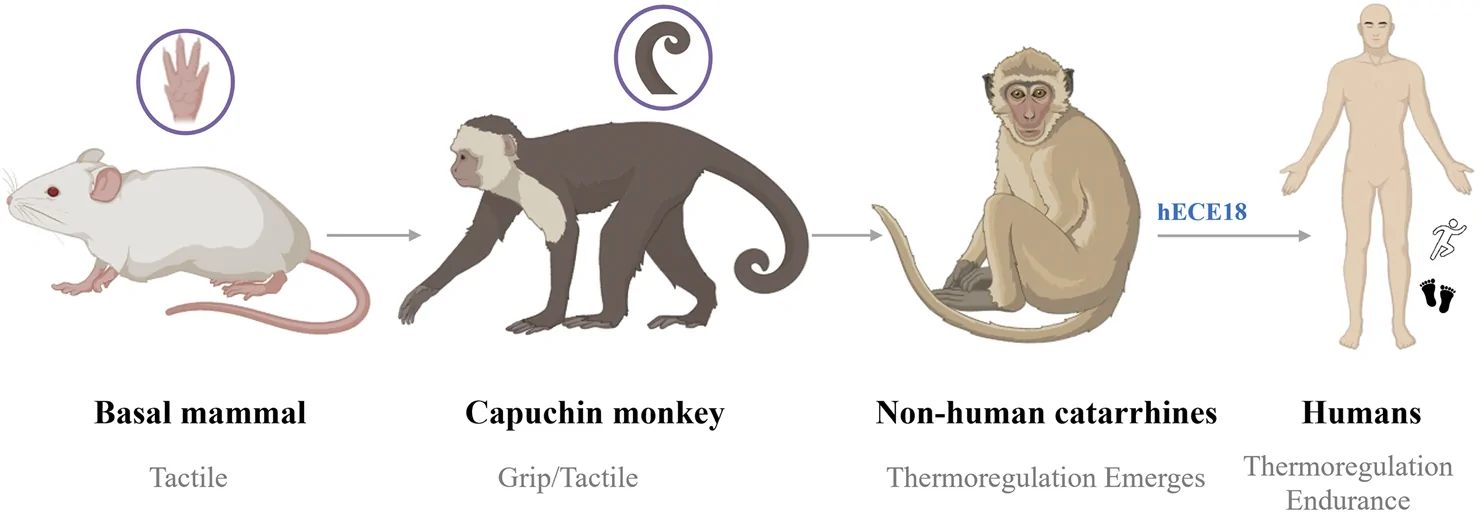
Evolutionary expansion of ESG distribution in primates. (Source: Created using templates from BioRender.com, based on phylogenetic distribution data from the cited primary literature). Rodents (e.g., mouse): ESGs confined to footpads (tactile/grip) (Taylor et al., 2012). New World monkeys (e.g., capuchin): ESGs expand to ventral tail tip (prehensile grip) (Best and Kamilar, 2018). Catarrhines (e.g., macaque): Body-wide ESG distribution emerges (thermoregulation) (Best and Kamilar, 2018). Humans: High-density body-wide ESGs (efficient evaporative cooling), driven by innovations including the human-specific enhancer hECE18, which has been linked to increased eccrine gland density (Aldea et al., 2021).

A major evolutionary shift took place within catarrhine primates (Old World monkeys, apes, and humans), which acquired widespread ESG distribution over the entire integument, thereby enabling efficient evaporative cooling and thermoregulation (Ellis and Montagna, 1962; Montagna and Yun, 1963) (Figure 2). The human lineage, while retaining this fundamental catarrhine blueprint, underwent an exceptional quantitative leap: with 2–4 million ESGs, humans exhibit a density roughly 5 to 10 times that of other Old World primates, which typically possess only 10%–20% of human ESG density (Kamberov et al., 2018) (Figure 2). This dramatic expansion is estimated to have emerged approximately 3 million years ago in East African savannas, driven by the thermoregulatory challenges of open habitats, the metabolic demands of endurance locomotion, and the increasing heat load associated with encephalization. This extraordinary density, combined with the evolutionary reduction of body hair, underpins human endurance running by creating a powerful thermoregulatory synergy: reduced hair minimizes insulation, while increased sweat glands maximize evaporative cooling efficiency (Bramble and Lieberman, 2004; Ruxton and Wilkinson, 2011).

### 2.6 EN1 as a quantitative rheostat and its regulatory control

The strict, dose-dependent relationship between *EN1* and ESG density is convincingly demonstrated by genetic experiments. *En1* heterozygotes exhibit a quantifiable reduction in ESG counts: on a C57BL/6 background, density drops from 13 ± 3 in wild-type controls to 5 ± 1 (a ∼60% decrease); on an FVB/N × C57BL/6 F1 hybrid background, from 40 ± 5 to 17 ± 8 (Kamberov et al., 2015). This dose effect is most evident in bipotential inter-footpad regions, supporting the model that *EN1* acts as a rheostat that balances hair follicle versus ESG fate (Kamberov et al., 2015; Lu et al., 2016). However, EN1 heterozygosity may affect additional uncharacterized targets, and the observed phenotype—particularly the reduced ESG density—could be influenced by indirect effects beyond the simple ESG-HF balance or by compensatory mechanisms. Other developmental pathways may also contribute to the phenotypic variation (Kamberov et al., 2015; Loomis et al., 1998; Murcia et al., 2004). Notably, natural variation in ESG density among mouse strains maps to cis-regulatory polymorphisms that affect *En1* expression levels, providing a genetic precedent for the regulatory evolution observed in primates (Kamberov et al., 2015).

This precedent points to the importance of understanding the specific regulatory inputs that modulate *EN1* expression. The precise level of *EN1* expression is controlled by a multi-layered regulatory network, the evolution of which explains the increased number of ESGs per unit area in human skin. As exemplified by the *Maenli*/LSEE1/2 hub in limb patterning (Section 2.3), such CRE-based regulation also operates in the context of ESG development. DEAF1 acts as a direct activator of *EN1*; DEAF1 binds conserved sites upstream of *EN1*, and *Deaf1* knockout mice phenocopy a partial *En1* loss-of-function, showing reduced ESG density and increased HFs in inter-footpad skin (Aldea et al., 2019). Within the *EN1* topologically associating domain (TAD), a modular enhancer network fine-tunes expression. ECE20 is a conserved, pleiotropic enhancer required for *EN1* expression in both the skin ectoderm and the midbrain; its knockout reduces *En1* by ∼20% and ESG number by ∼30% (Aldea et al., 2023). In contrast, ECE18 is a primate-specific, rapidly evolved enhancer. The human version (hECE18) contains 12 lineage-specific mutations. Among these, mutations at two specific sites (termed SP1^A^ and SP1^B^) were shown to create high-affinity SP1 binding sites that contribute to its enhanced activity (Aldea et al., 2021). In quantitative luciferase reporter assays in cultured human keratinocytes, hECE18 produced on average a 13-fold increase in luciferase activity over control (Aldea et al., 2021). CRISPR interference (CRISPRi)-mediated repression of hECE18 reduces endogenous *EN1* by ∼40%, and knock-in of hECE18 into mice elevates ectodermal *En1* expression and increases ESG density (Aldea et al., 2021). This modular network—a conserved, pleiotropic core (ECE20) and a recently co-opted, tissue-specific fine-tuner (ECE18)—allowed for the dramatic, yet non-lethal, increase in human ESG density during evolution (Aldea et al., 2021). However, the relative contribution of each enhancer to total EN1 expression across different anatomical sites and developmental stages remains to be quantitatively assessed, particularly in human tissues.

## 3 Development of eccrine sweat glands and the role of EN1

ESG development proceeds through a precisely orchestrated sequence of epithelial-mesenchymal interactions, with EN1 at its apex (Cui and Schlessinger, 2015; Lu et al., 2016) (Figure 3). Understanding this developmental cascade is essential for interpreting the tiered pathogenesis model proposed in Section 4.

**FIGURE 3:**
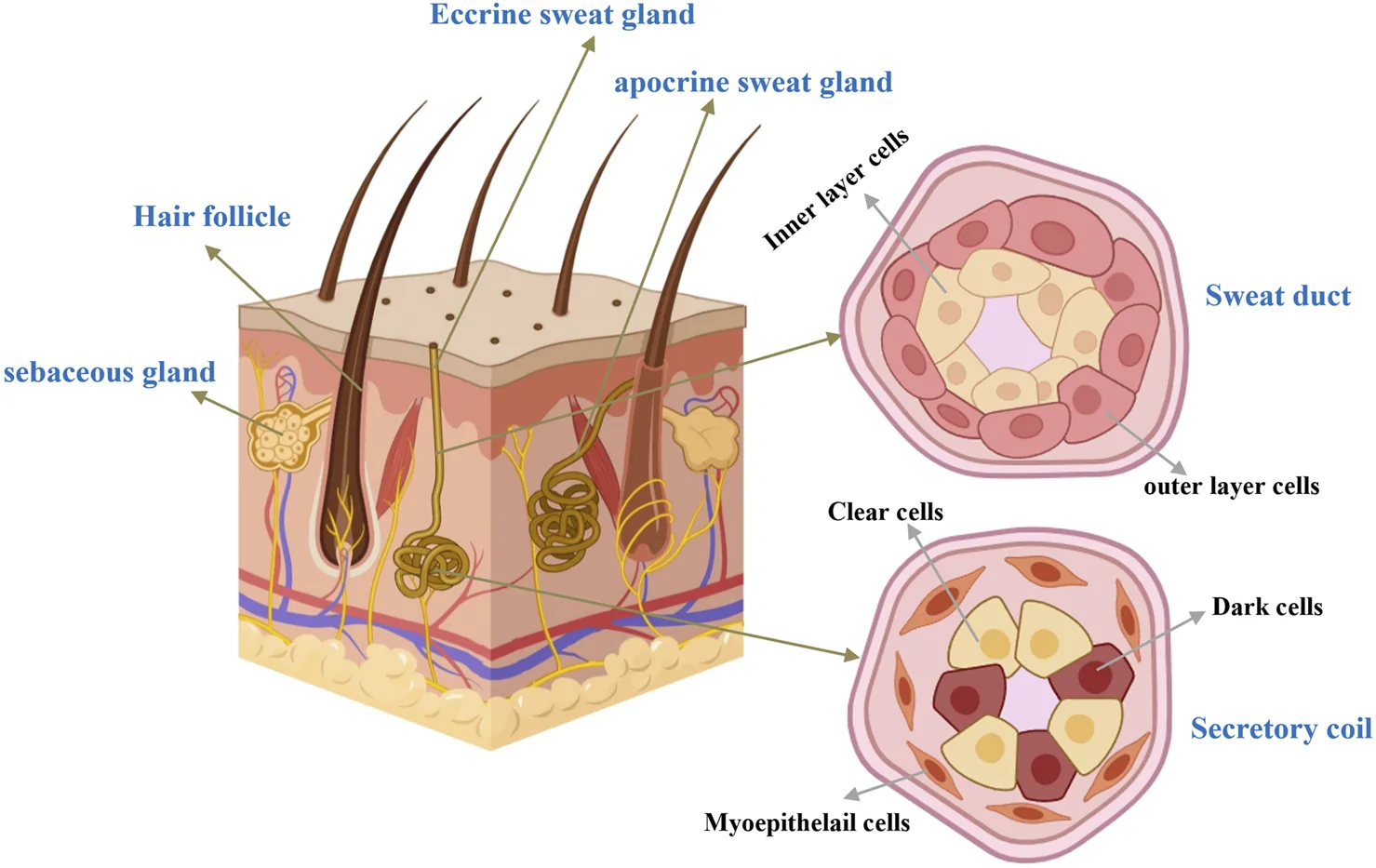
Anatomy and cellular organization of human ESGs (Source: Created using templates from BioRender.com). (Left) Schematic diagram of human skin architecture, depicting the three primary layers—epidermis, dermis, and hypodermis—and major appendages. The ESG is a simple coiled tubular gland consisting of a deep secretory portion and a duct that opens directly onto the skin surface. Other illustrated structures include the pilosebaceous unit (hair follicle and associated sebaceous gland) and the apocrine sweat gland, which typically connects to a hair follicle (Lin et al., 2021; Saga, 2002). (Right, Top) Cross-sectional view of the ductal segment, composed of an inner layer of ductal cells and an outer layer of ductal cells (Lin et al., 2021; Saga, 2002). (Right, Bottom) Cross-sectional view of the secretory coil, showing the three principal cell types: clear cells, dark cells, and surrounding myoepithelial cells (Lin et al., 2021; Saga, 2002).

### 3.1 Structure of eccrine sweat glands

ESGs are simple, coiled tubular glands, each consisting of a deep secretory coil and a ductal system (Saga, 2002; Sato et al., 1989) (Figure 3). The secretory coil contains 3 cell types: clear cells (produce watery fluid), dark cells (secrete glycoproteins), and myoepithelial cells (contractile). The duct reabsorbs sodium and chloride to produce hypotonic sweat, which is released onto the skin surface through the acrosyringium (Cui and Schlessinger, 2015; Lin et al., 2021) (Figure 3).

### 3.2 Morphogenetic cascade of ESGs

The morphogenetic cascade of ESGs is a tightly orchestrated, multi-stage process driven by sequential and reciprocal epithelial-mesenchymal signaling interactions. This cascade proceeds through five distinct phases, beginning with the establishment of ventral identity and culminating in functional maturation of the secretory apparatus.

#### 3.2.1 The ventral permissive field

In mice and rats, ESGs are restricted to the footpads, which are derived from the ventral aspect of the developing limb; in humans, the homologous glabrous skin is found on the palms and soles. This anatomical restriction is established early in embryogenesis by EN1, which represses the dorsal determinant *Wnt7a* in the ventral ectoderm, thereby specifying ventral identity and facilitating the proper formation of the Apical Ectodermal Ridge (Loomis et al., 1996; Tsugane and Yasuda, 1998). Through this function, EN1 creates a ventral domain that is anatomically and molecularly competent for ESG development—at least in species where ESGs are confined to ventral limb derivatives. The necessity of EN1 for establishing this permissive field is underscored by the *En1* knockout phenotype: ventral footpads are transformed into hair-bearing, dorsal-type epidermis, and ESGs are completely absent (Loomis et al., 1996; Wurst et al., 1994).

#### 3.2.2 Pre-placode patterning

Within this ventrally specified field, the developmental landscape of the skin is further pre-patterned to define regions competent for ESG formation. This pre-placode stage is characterized by the establishment of an ESG-permissive dermal microenvironment, a process governed by intrinsic regional genetic codes (Cui and Schlessinger, 2015). In mice, dermal fibroblasts in ESG-prone ventral skin (e.g., footpads) are intrinsically programmed to constitutively express BMPs (such as BMP5), FGFs (including FGF2, FGF7, and FGF18), and Wnt ligands (Lu et al., 2016; Plikus et al., 2004) (Figure 4). The secretion of these factors creates an instructive niche that pre-defines the sites for future appendage formation (Lu et al., 2016; Plikus et al., 2004). The dermis of the ventral footpad, characterized by high BMP and FGF signaling, signals to the overlying epithelium to suppress SHH, a key inducer of hair follicle fate, thereby creating a molecular environment permissive for eccrine development (Lu et al., 2016). EN1 is expressed in the responding ventral epidermal progenitors and is required for executing this glandular program (Dingwall et al., 2024; Lu et al., 2016).

**FIGURE 4:**
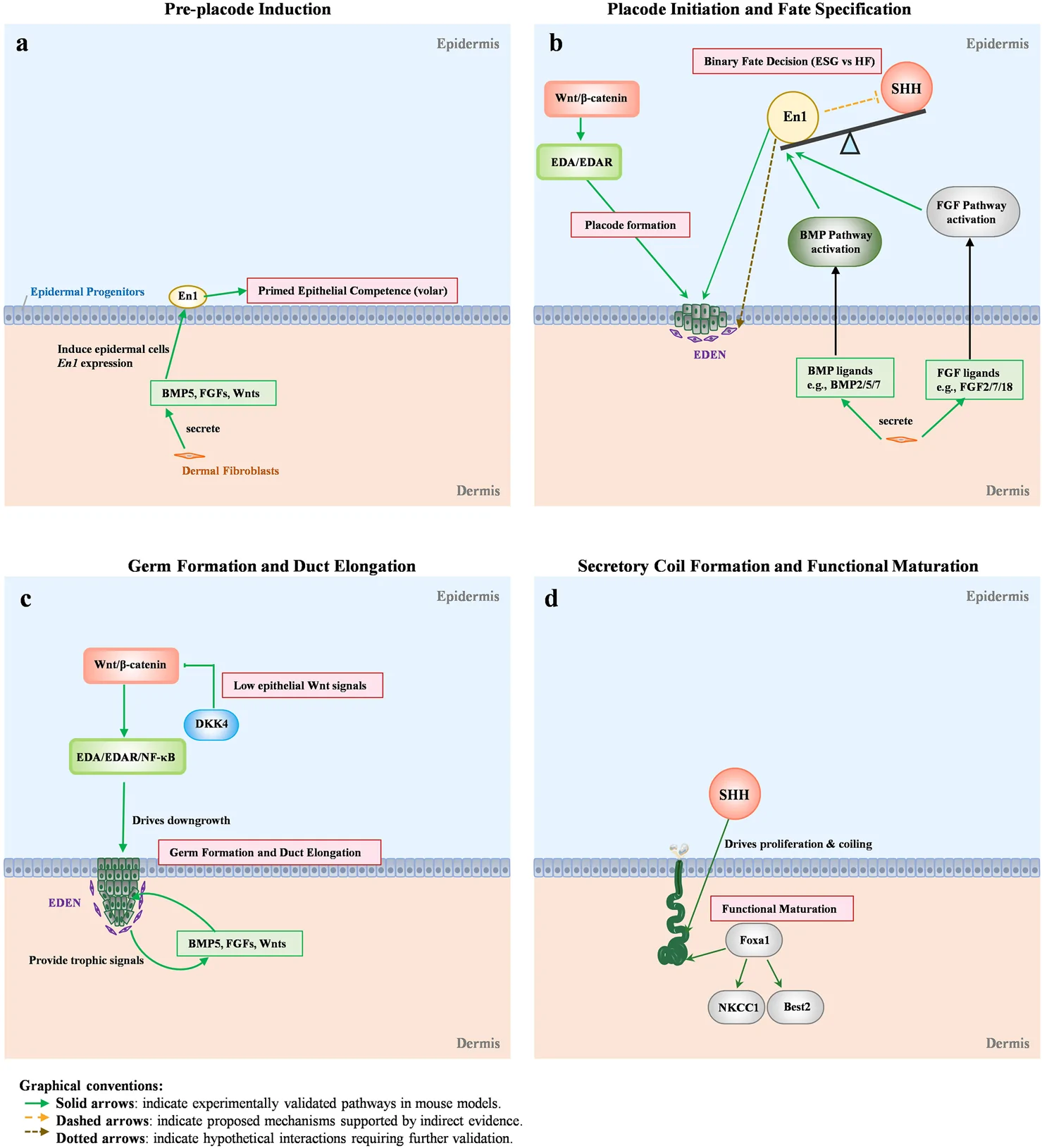
Proposed model for the Stage-specific signaling cascade governing ESG morphogenesis. (Source: Authors; Created with Microsoft PowerPoint based on synthesized data from the cited primary literature.) The schematic illustrates four consecutive developmental stages with corresponding morphological changes and stage-specific molecular signaling pathways. **(a)** Pre-placode Induction. Dermal fibroblasts in prospective ESG regions constitutively express BMP5, FGFs, and Wnt ligands, creating an instructive niche that primes the overlying epithelium for ESG fate specification (Cui and Schlessinger, 2015; Lu et al., 2016). **(b)** Placode Induction and Fate Specification. Epithelial Wnt/β-catenin activation initiates placode formation and upregulates EDA/EDAR/NF-κB and its feedback inhibitor DKK4. Dermal BMP and FGF signals converge on the placode, creating a signaling environment (high BMP/FGF, low SHH) that is permissive for EN1 function. EN1 is proposed to commit the placode to the sweat gland lineage, in part enabling the repression of *Shh*, while simultaneously proposed to induce the formation of the EDEN beneath the placode (Cui et al., 2014; Dingwall et al., 2024; Kamberov et al., 2015; Lu et al., 2016). **(c)** Germ Formation and Duct Elongation. EDA/EDAR/NF-κB signaling drives epithelial downgrowth, supported by the EDEN, which forms a protective sheath and provides paracrine signals (WNTs, BMPs, FGFs). Wnt/β-catenin signaling is suppressed by sustained Dkk4 expression (Cui et al., 2014; Dingwall et al., 2024; Lu et al., 2016). **(d)** Coil Formation and Maturation. SHH signaling becomes essential for the subsequent proliferative expansion and coiling of the secretory coil. FOXA1 directs functional maturation of dark cells by regulating Best2 and NKCC1. The EDEN regresses, and EDA/EDAR and epithelial Wnt pathways are inactivated, marking completion of morphogenesis (Cui et al., 2012; Cui and Schlessinger, 2015; Cui et al., 2014; Dingwall et al., 2024). Graphical conventions: Solid arrows indicate experimentally validated pathways in mouse models; dashed arrows indicate proposed mechanisms supported by indirect evidence; dotted arrows indicate hypothetical interactions requiring further validation. Abbreviations: ESGs, eccrine sweat glands; SHH, Sonic Hedgehog; EN1, Engrailed-1; EDEN, EN1-dependent eccrine niche; BMP, Bone Morphogenetic Protein; FGF, fibroblast growth factor; NKCC1, Na^+^-K^+^-2Cl^-^ cotransporter 1; Best2, Bestrophin 2.

#### 3.2.3 Placode initiation and binary fate specification

The transition from a broad pre-patterned field into discrete organ rudiments begins with focal upregulation of canonical Wnt/β-catenin signaling within clusters of basal epidermal cells. Nuclear β-catenin and its co-factor LEF1 become concentrated in these nascent placodes, defining the precise anatomical locations of future sweat germs (Cui et al., 2014) (Figure 4). Genetic ablation of epithelial β-catenin completely abolishes placode formation, establishing the indispensability of this pathway for initiation (Cui et al., 2014). Downstream, Wnt/β-catenin activates key targets including *Edar* and the secreted antagonist *Dkk4*, which operates in a negative feedback loop to finely balance Wnt activity and determine the spatial density of gland induction (Cui et al., 2014).

Concurrently, while Wnt signaling defines “where” a placode forms, the instructive BMP signal from the dermis dictates “what” it becomes, an ESG or a hair follicle (Lu et al., 2016; Plikus et al., 2004). In mice, conditional knockout of the epithelial BMP receptor *Bmpr1a* in the footpad completely abolishes ESG development and redirects the progeny toward HF-like structures, while inhibition of BMP signaling can reprogram ESG buds toward an HF fate (Lu et al., 2016). A core conserved mechanism downstream of elevated BMP/FGF signaling is the active suppression of *Shh*, the canonical HF inducer (Lu et al., 2016). In mouse ventral foot skin, *Shh* expression is conspicuously absent, whereas it is robustly expressed in dorsal skin placodes. Forced *Shh* expression in the ventral footpad suppresses ESG formation and induces HF fate, confirming a mutual antagonism: high BMP/FGF activity represses *Shh* to permit ESG fate, while SHH signaling inhibits the ESG program to promote folliculogenesis (Lu et al., 2016).

EN1 is required for the repression of *Shh* in the ventral ectoderm, as genetic ablation of *En1* leads to ectopic *Shh* activation and a subsequent switch to a HF fate (Lu et al., 2016). However, whether EN1 represses *Shh* through direct DNA binding or via indirect transcriptional mechanisms remains an open question. Single-cell RNA-seq has confirmed that *En1*+ progenitors follow a distinct differentiation trajectory, characterized by upregulation of *Dkk4*, *Wnt10a*, and *Eda* (Dingwall et al., 2024). Concurrently with fate specification, epidermal EN1 is required for the specification of S100a4-positive fibroblasts in the adjacent dermis, which form the EDEN and provide essential inductive support for subsequent morphogenesis in mice (Dingwall et al., 2024). However, whether this specification is mediated by direct (secreted EN1) or indirect (EN1-induced secondary signals) mechanisms remains unresolved.

#### 3.2.4 Germ Formation and duct elongation

Following fate specification, the EDA/EDAR/NF-κB pathway drives the downgrowth and elongation of the epithelial germ into the dermis (Cui et al., 2009; Cui et al., 2014) (Figure 4). In *Eda*-null mice, although *Lef1*-positive placodal clusters form, they fail to properly invaginate, do not elongate into ducts, and eventually regress via apoptosis (Cui et al., 2014). During this phase, the EDEN matures and becomes functionally indispensable. Lineage-tracing studies demonstrate that S100a4-positive fibroblasts give rise to the dermal sheath cells enveloping the elongating epithelial germ, providing rich bidirectional signaling that is essential for successful ductal morphogenesis in mouse models (Branch and Ezhkova, 2024; Dingwall et al., 2024). Genetic ablation of the S100a4^+^ cell population leads to a ∼45% reduction in ESG formation, demonstrating that the EDEN provides essential trophic, mechanical, and guidance support for downgrowth (Dingwall et al., 2024).

#### 3.2.5 Secretory coil formation and functional maturation

Following duct formation, SHH signaling becomes essential for the subsequent proliferative expansion and coiling of the secretory coil (Cui et al., 2014) (Figure 4). Its specific ablation permits normal germ induction and duct elongation but severely impairs secretory coil development due to reduced cell proliferation (Cui et al., 2014; Huang et al., 2016). Terminal functional differentiation is then governed by the transcription factor FOXA1, which regulates key ion transporters like Bestrophin 2 and NKCC1, essential for sweat secretion (Cui et al., 2012). *Foxa1* ablation in mice results in complete anhidrosis despite continued gland morphogenesis, accompanied by downregulation of *Nkcc1* and *Best2* as well as glycoprotein accumulation in gland lumens and ducts (Cui et al., 2012). Concurrently, cellular differentiation establishes the bilayered secretory coil, with K8/K18-positive luminal cells enveloped by α-smooth muscle actin-positive myoepithelial cells that provide contractile force (Saga, 2002; Sato et al., 1989). At this stage, the EDEN has regressed, and the earlier dominant EDA/EDAR and epithelial Wnt pathways are inactivated (Branch and Ezhkova, 2024; Dingwall et al., 2024). The stage-specific roles of EN1 during ESG morphogenesis are summarized in Table 2.

**TABLE 2:** Stage-specific roles of EN1 in ESG morphogenesis.

Developmental stage	Morphological event	Key signaling pathways	EN1 role	Evidence level	Experimental evidence	References
Limb field establishment	Ventral ectoderm specification; glabrous skin territory	EN1 → repression of WNT7A/LMX1B	Creates permissive ventral field for ESG development	Definitive	*En1* KO (mouse): limb dorsalization, loss of footpads, absence of ESGs	( Loomis et al., 1996; Wurst et al., 1994 )
Pre-placode patterning	Dermal fibroblast niche specification; epidermal competence	BMP5, FGFs, Wnts	Expressed in ventral epidermal progenitors in response to the dermal BMP/FGF-rich microenvironment; EN1 is required downstream of these signals to execute the glandular program and repress the HF fate	Strong	Dermal fibroblast signaling induces epithelial *En1*	( Lu et al., 2016; Plikus et al., 2004 )
Placode initiation	Focal Wnt/β-catenin activation	Wnt/β-catenin, EDA/EDAR	EN1+ progenitors commit to ESG fate	Correlative	EN1 protein detected in nascent placodes (IHC); direct functional evidence lacking	(Mainguy et al., 1999)
Fate specification	Binary ESG vs. HF decision	BMP/FGF vs. SHH	Promotes glandular program; direct EN1 occupancy remains unconfirmed	Definitive	*Bmpr1a* KO: ESG→HF conversion; forced Shh: ESG suppression	(Lu et al., 2016)
EDEN induction	S100a4^+^ fibroblast niche formation	EN1-dependent paracrine signals	Non-cell-autonomous niche induction; molecular cue remains unresolved	Strong	EDEN ablation (*S100a4*- DTR): ↓∼45% ESG reduction despite persistent *En1*	(Dingwall et al., 2024)
Germ formation	Epithelial downgrowth into dermis	EDA/EDAR; EDEN-derived signals	EN1 expression maintained; EDEN provides support for invagination	Definitive	EN1 expression persists in downgrowing germs (IHC); EDEN ablation leads to abortive downgrowth	(Dingwall et al., 2024; Mainguy et al., 1999)
Duct elongation	Duct formation and extension	EDEN-derived trophic signals	EN1 expression sustained in ductal cells	Definitive	EN1 protein in ductal cells (IHC); EDEN ablation leads to abortive gland downgrowth and reduced ESG density	( Dingwall et al., 2024; Mainguy et al., 1999 )
Adult maintenance	Structural integrity (proposed)	EN1-BPAG1e axis (*in vitro*)	EN1 expressed in adult volar keratinocytes; proposed role in adult gland maintenance remains untested	Speculative	Engrailed proteins bind the *Bpag1e* promoter (EMSA/DNase I footprinting) and regulate it in cultured keratinocytes; conditional *En1* ablation in adult epidermis not reported	(Mainguy et al., 2000)

Abbreviations: BMP, bone morphogenetic protein; DTR, diphtheria toxin receptor; EDEN, EN1-dependent eccrine niche; ESG, eccrine sweat gland; FGF, fibroblast growth factor; HF, hair follicle; IHC, immunohistochemistry; KO, knockout; SHH, sonic hedgehog.

Evidence Level Definitions: Definitive = multiple lines of evidence (genetic + biochemical + functional) validated *in vivo*, with cross-species validation where available; Strong = primarily from a single model system (e.g., mouse), lacking cross-species validation; Correlative = expression or phenotypic association without functional validation; Speculative = theoretical inference, no direct experimental evidence.

### 3.3 EN1 as a central regulator for ESG lineage specification

EN1 serves as a critical node in the specification of ESGs. Its necessity is established by loss-of-function studies: En1 null mice display a complete absence of ESGs, with ventral footpad epithelia adopting a hair-bearing, dorsal-like identity (Loomis et al., 1996; Wurst et al., 1994). Conversely, ectopic *En1* activation in dorsal skin, when combined with elevated BMP/FGF signaling, suppresses HF markers and induces glandular features, supporting its sufficiency in promoting eccrine fate under permissive mesenchymal signals (Lu et al., 2016). Beyond this qualitative control, EN1 also acts in a dose-dependent manner; even heterozygous reduction of *En1* significantly reduces ESG density, underscoring its quantitative role in appendage specification (Kamberov et al., 2015).

Recent single-nucleus transcriptomic profiling has uncovered an EN1-dependent, eccrine-enriched transcriptional signature in developing ESGs. Among the few genes differentially expressed between eccrine and hair placodes, *Dkk4*—a secreted Wnt inhibitor—stood out as a candidate mediator of EN1-dependent fate specification (Dingwall et al., 2024). The upregulation of *Dkk4* may reinforce the fate-specifying effects of the established BMP–SHH antagonism, in which mesenchymal BMP signaling suppresses epithelial *Shh* expression to permit eccrine development (Lu et al., 2016). Notably, while EN1 is required for *Shh* repression in the ventral ectoderm, direct transcriptional regulation of *Shh* by EN1 has not been demonstrated. Instead, dermal signals such as *Bmp5* and *Fgf18* have been characterized as upstream inducers of *En1* expression in the ventral limb ectoderm (Lu et al., 2016). At the other end of the differentiation cascade, terminal secretory function—including expression of BEST2, NKCC1, and AQP5—is governed by downstream transcription factors such as FOXA1 rather than by direct EN1 occupancy at these loci (Cui et al., 2012). This observation suggests a sequential relay model, in which EN1 may act early to establish chromatin permissiveness or signaling context, while subsequent, EN1-independent cascades execute the secretory machinery.

Despite these advances, several key questions remain unresolved. First, although EN1 is required for *Shh* repression in the ventral footpad, the genetic epistasis between EN1 and BMP/FGF pathways has not been defined—it is unclear whether EN1 acts downstream of these signals to repress *Shh*, or whether the pathways converge independently. Second, the precise threshold and duration of BMP/FGF signaling needed to initiate and sustain *En1* expression have not been determined, nor has the possibility of reciprocal reinforcement been formally tested (Dingwall et al., 2024). Third, the potential functional redundancy between *En1* and *En2* in ESG development remains unexplored; although *En2* is expressed in the nervous system, its expression in ventral limb ectoderm has not been systematically examined, and *En2* cannot substitute for *En1* in limb patterning (Hanks et al., 1998). Fourth, as summarized in Table 1, direct binding assays (e.g., ChIP-seq) have not been reported in eccrine-competent epidermis, leaving open whether EN1 acts directly at target loci or indirectly through secondary transcription factors and chromatin remodelers.

Beyond these core issues, other signaling modules may intersect with EN1 function. In fibroblasts, mechanical tension activates EN1 via YAP, and EN1 in turn amplifies TGF-β/SMAD3 signaling to promote fibrosis (Mascharak et al., 2021). Whether analogous crosstalk occurs in epithelial progenitors during ESG development remains unknown, as direct evidence linking pathways such as Notch, YAP/TAZ, or other Hedgehog components (PTCH1, SMO, GLI) to EN1-dependent specification is currently lacking (Cui et al., 2014; Elbediwy et al., 2016; Nowell and Radtke, 2013; Rognoni and Walko, 2019; Woo et al., 2012).

### 3.4 EN1 orchestrates the EDEN: a working model

The EDEN exemplifies a non-cell-autonomous function of EN1. Epidermal EN1 is required for the specification of S100a4^+^/Tnc^+^fibroblasts in the underlying dermis (Dingwall et al., 2024). However, it is unknown whether this inductive event is mediated by a secreted EN1 protein, by an EN1-induced secondary paracrine signal, or through other indirect mechanisms. While EN1 possesses the intrinsic capacity for unconventional secretion in cell culture systems (Joliot et al., 1998; Maizel et al., 2002), its functional significance for EDEN induction *in vivo* remains a compelling but untested hypothesis. This transient niche, detected in human fetal plantar skin at 120 gestational days, is absent around mature eccrine glands in adult cheek skin (Dingwall et al., 2024). Whether a similar S100a4^+^ population persists or regresses around adult palmar/plantar eccrine glands remains to be systematically examined. CellChat analysis of snRNA-seq data from mouse volar skin predicts rich bidirectional signaling between EDEN fibroblasts and the developing eccrine epithelium, involving Wnt, BMP, and TGF-β pathways (Dingwall et al., 2024). Functional validation using diphtheria toxin-mediated ablation of S100a4^+^ cells reduced ESG density by ∼45% despite persistent epidermal *En1* expression, demonstrating its necessity for gland morphogenesis in mice (Dingwall et al., 2024). The identification of EDEN resolves a long-standing question in skin biology: how eccrine glands, which lack a condensed dermal papilla like that of hair follicles, nonetheless receive the mesenchymal support required for their development.

Nevertheless, the precise molecular nature of the EN1-derived cue that initiates EDEN specification remains unresolved. While EN1 possesses the intrinsic capacity for unconventional secretion (see Section 2.2), the EDEN-inducing activity could also be mediated by secondary paracrine factors upregulated cell-autonomously in EN1+ epithelial cells. Distinguishing between these direct and indirect mechanisms awaits lineage-specific tools that abrogate EN1 secretion without affecting its transcriptional function—a tool yet to be developed. Until then, the EDEN induction model should be regarded as a compelling hypothesis rather than a proven *in vivo* paradigm (see Table 1). Moreover, whether a comparable transient dermal niche exists during human ESG development, and whether it is induced by the same EN1-dependent cues, remains to be determined (see Table 3).

**TABLE 3:** Cross-species summary of EN1 function in ESG development and maintenance.

Evidence Category	Mouse Evidence	Human Evidence
EN1 expression in ventral ectoderm/ESG progenitors	Definitive (mouse models): ISH and IHC in embryonic ventral limb ectoderm and footpad placodes ( Loomis et al., 1996; Mainguy et al., 1999 )	Validated (human tissues): EN1 expression was detected by qRT-PCR in isolated epithelial buds from fetal scalp and palm (Lu et al., 2016 )
EN1 necessity for ESG specification and formation	Definitive (mouse models): *En1* KO causes limb dorsalization, loss of footpads, and complete ESG absence ( Loomis et al., 1996; Lu et al., 2016; Wurst et al., 1994 )	Critical Gap (human tissues): no human EN1 nulls reported
EN1 as a dose-dependent rheostat for ESG density	Definitive (mouse models): heterozygotes show ↓∼60% ESG density ( Kamberov et al., 2015 )	Partial (comparative evolutionary studies and human cells): hECE18 activity validated in human keratinocytes using luciferase reporter assays (13-fold↑ luciferase) and CRISPRi-mediated repression (↓∼40% EN1); human *in vivo* proof lacking (Aldea et al., 2021 )
EN1 as binary fate switch (ESG vs. HF)	Definitive (mouse models): gain- and loss-of-function in mice ( Lu et al., 2016 )	Partial (comparative evolutionary studies): Inferred from human genetic variation ( Lu et al., 2016 )
EN1 downstream targets	Correlative (mouse models): snRNA-seq identified *Dkk4* as an En1-dependent, eccrine-enriched gene (Dingwall et al., 2024 )	Critical Gap: Human data lacking
EN1-dependent EDEN specification and requirement	Strong (mouse model): *S100a4* ^*+*^ */Tnc* ^*+*^ fibroblasts; ablation → ↓∼45% ESG formation ( Dingwall et al., 2024 )	Partial (human tissues): EDEN markers (*S100A4/TNC*) present in human fetal glabrous skin; EN1-dependence not tested ( Dingwall et al., 2024 )
EN1 as a candidate regulator of BPAG1 and adult ESG maintenance	Speculative (*in vivo*): Engrailed proteins regulate the *Bpag1e* promoter in cultured cells (EMSA/DNase I footprinting); adult gland maintenance function untested *in vivo* ( Mainguy et al., 1999; Mainguy et al., 2000 )	Validated (human cells)/Not tested (*in vivo*): Engrailed proteins regulate the *BPAG1e* promoter in human keratinocyte cell lines; adult gland maintenance function untested *in vivo* ( Mainguy et al., 1999; Mainguy et al., 2000 )
EN1 enhancer evolution (ECE18/ECE20)	Definitive (mouse models): *Ece20* KO → ↓∼20% En1, ↓∼30% ESG; ECE18 absent in mouse genome ( Aldea et al., 2023 )	Validated (human cells): Activity of ECE18 validated using luciferase reporter assays and CRISPRi-mediated repression in cultured human keratinocytes (13-fold↑ luciferase; CRISPRi ↓∼40% EN1) Critical Gap: ECE20 — human *in vivo* validation lacking (Aldea et al., 2021; Aldea et al., 2023 )
EN1 in fibrosis/scarring	Definitive (mouse models): EPFs drive scarring; fibroblast-specific *En1* KO → ↓fibrosis ( Mascharak et al., 2021; Rinkevich et al., 2015 )	Partial (human tissues): EN1 re-expressed in SSc fibroblasts) ( Györfi et al., 2021; Mascharak et al., 2021 )

Abbreviations: ChIP, chromatin immunoprecipitation; EPF, Engrailed-1 positive fibroblast; ESG, eccrine sweat gland; HF, hair follicle; hECE18, human EN1 candidate enhancer 18; IHC, immunohistochemistry; ISH, *in situ* hybridization; KO, knockout; SSc, systemic sclerosis. Mouse Evidence Level Definitions: Definitive = multiple lines of evidence (genetic + biochemical + functional) validated *in vivo*; Strong = primarily from a single model system, with functional validation but lacking comprehensive genetic and biochemical evidence (e.g., no conditional knockout, no direct binding data, or evidence from a single approach); Correlative = expression or phenotypic association without functional validation; Speculative = theoretical inference, no direct experimental evidence. Human Evidence Level Definitions: Validated = confirmed in human tissues/cells; Partial = some human data available but gaps remain; Critical Gap = no functional human validation; Not tested = no human data available.

### 3.5 EN1 as a candidate regulator of BPAG1e in adult skin: implications for ESG maintenance

Following the completion of morphogenesis and regression of the EDEN, EN1 expression is maintained in adult volar keratinocytes, including those associated with eccrine tubules, as demonstrated by LacZ reporter activity in *En*1^lki/*+*^ mouse footpads (Mainguy et al., 1999). Among the candidate downstream effectors through which EN1 might influence adult gland integrity is BPAG1 (bullous pemphigoid antigen 1), a plakin-family cytoskeletal linker protein with tissue-specific isoforms.

Using DNase I footprinting and electrophoretic mobility shift assays (EMSA) with the Engrailed homeodomain (EnHD, derived from En2), Mainguy et al. showed that Engrailed proteins bind directly to conserved regulatory elements within the *Bpag1e* promoter *in vitro* (Mainguy et al., 1999). In co-transfection experiments using a *Bpag1e* promoter–luciferase reporter in human keratinocytes, full-length En2 repressed promoter activity, whereas an N-terminally truncated En2 enhanced it, indicating context-dependent transcriptional regulation (Mainguy et al., 1999). A subsequent gene-trap screen further identified the *BPAG1* locus as a candidate Engrailed-regulated target (Mainguy et al., 2000). Although these experiments employed En2 rather than EN1, the homeodomains are highly conserved between the two proteins. Based on this homology and the sustained expression of EN1 in adult volar epidermis and eccrine tubules, EN1 has been proposed as a candidate regulator of BPAG1e in adult skin (Mainguy et al., 1999).

However, these regulatory relationships have been established exclusively *in vitro*. Direct EN1 occupancy at the *BPAG1e* locus *in vivo* has not been demonstrated, and conditional ablation of *En1* in adult epidermis has not been reported. Therefore, the proposed role of this axis in maintaining adult eccrine gland integrity remains a hypothesis that awaits experimental validation *in vivo*.

Beyond its epithelial adhesion function, BPAG1 isoforms have additional roles. Loss of the neural isoform BPAG1n, for instance, has been associated with degeneration of sympathetic neurons and reduced autonomic nerve fiber density around sweat glands in *dt/dt* mice (Tseng et al., 2011). This neuronal phenotype, however, reflects an isoform-distinct mechanism and does not provide evidence for a role of epithelial BPAG1e in sweat gland innervation or maintenance.

## 4 Regeneration of eccrine sweat glands: a tiered pathogenesis–regeneration model

The conceptual model of EN1 function presented here offers a working framework for thinking about regenerative strategies. By defining the specific level at which the developmental program fails, we can design targeted interventions. We propose a tiered model as a conceptual approach to link pathogenesis to potential therapeutic strategies (Table 4; Figure 5). However, several critical caveats must be explicitly stated at the outset.

**TABLE 4:** Tiered pathogenesis–regeneration model for EN1-Dependent eccrine sweat gland dysfunction.

Tier	Pathogenesis and consequence	Evidence level	Biomarker candidates	Regenerative strategy	Associated human conditions
Tier 1	Loss of ventral permissive field → Complete ESG agenesis with limb dorsalization ( Loomis et al., 1996; Wurst et al., 1994 )	Definitive (mouse)	Loss of *EN1*/*BMP7*; ectopic *WNT7A*/*LMX1B*	Bioengineered glabrous skin substitutes; *de novo* field reprogramming	Severe congenital dorsoventral patterning defects (inferred from mouse models); no pathogenic human *EN1* mutations have been confirmed to date
Tier 2	Failure of lineage specification → Reduced ESG density; partial HF transformation( Kamberov et al., 2015 ); hECE18 activity validated in human keratinocyte cell lines (13-fold↑ luciferase; CRISPRi ↓∼40% *EN1*) ( Aldea et al., 2021 ); *Deaf1* mutations → reduced *En1* and ESG number ( Aldea et al., 2019 )	Definitive (mouse)	Reduced *EN1* and/or *DKK4* (mouse-validated candidate) in glabrous skin	BMP4/5+FGF18+SHH inhibition; CRISPRa-*EN1*; AAV-*EN1*	No confirmed pathogenic human variants in EN1, DEAF1, or ECE18 have been reported to date. The genetic evidence is currently derived solely from mouse models and human cell-based assays
Tier 3	Impaired morphogenesis due to EDEN deficiency/absence → aborted gland downgrowth; immature/dysplastic glands (Dingwall et al., 2024). The molecular cue by which EN1 induces EDEN specification has not been resolved	Definitive (mouse)	Reduced *S100A4*/*TNC*; abortive gland downgrowth	EDEN-like fibroblast reprogramming; iPSC-derived niche; synthetic scaffolds	No human EDEN-deficient phenotypes have been characterized to date
Tier 4	Proposed EN1--BPAG1e regulatory axis supported by *in vitro* evidence showing that related EN2 can bind and regulate the *BPAG1e* promoter in cultured keratinocytes (Mainguy et al., 1999; Mainguy et al., 2000); direct evidence for EN1 regulation of BPAG1e *in vivo*, and its role in maintaining adult eccrine duct integrity, is entirely lacking	Correlative (*in vitro*)/Speculative (*in vivo*)	Diminished BPAG1e IHC; hemidesmosomal splitting on EM	AAV-*BPAG1e*; CRISPRa-*BPAG1e*; EN1 stabilizers/peptides	BPAG1e-related skin fragility; hypothesized mechanical ductal injury

Abbreviations: CRISPRa, CRISPR, activation; CRISPRi, CRISPR, interference; EDEN, EN1-dependent eccrine niche; EM, electron microscopy; ESG, eccrine sweat gland; FGF, fibroblast growth factor; HF, hair follicle; IHC, immunohistochemistry; iPSC, induced pluripotent stem cell; KO, knockout; TNC, Tenascin-C.

All regenerative strategies listed are conceptual and have not been validated in human ESG, regeneration.

Evidence Level definitions: Definitive = multiple lines of evidence (genetic + biochemical + functional) validated *in vivo*, with cross-species validation where available; Strong = primarily from a single model system (e.g., mouse), lacking cross-species validation; Correlative = expression or phenotypic association without functional validation; Speculative = theoretical inference, no direct experimental evidence.

**FIGURE 5:**
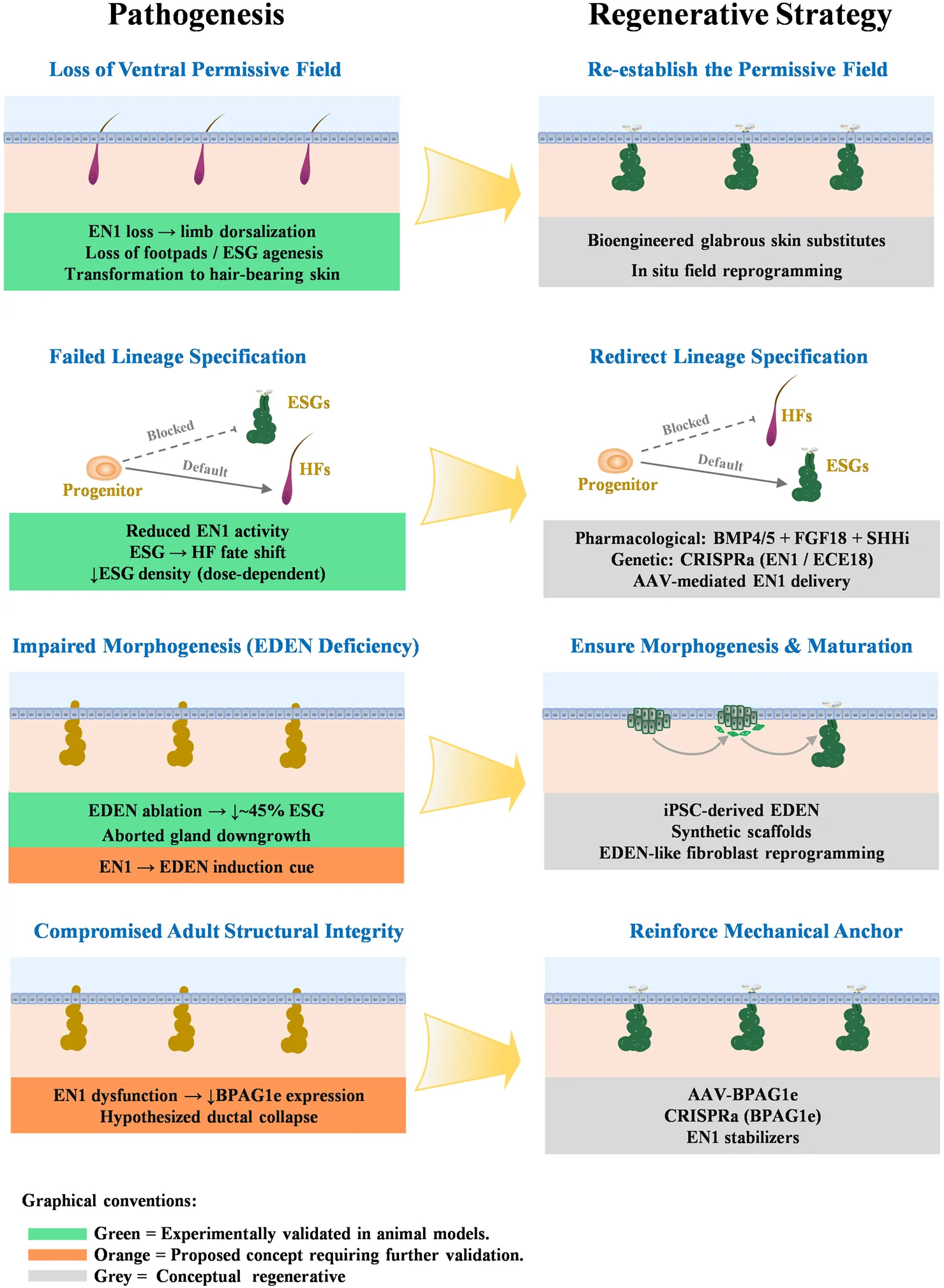
A conceptual tiered therapeutic framework for EN1-informed ESG regenerative strategies. (Source: Authors; Created with Microsoft PowerPoint based on synthesized data from the cited primary literature.) Tier 1: Loss of Ventral Permissive Field → Re-establish the Permissive Field (Loomis et al., 1998; Wurst et al., 1994). Pathogenesis: Skin lacks ventral (glabrous) identity and is dominated by dorsal/follicular programs; ESGs are completely absent.Regenerative Strategy: Bioengineered glabrous skin substitutes; *de novo* field reprogramming. Tier 2: Failed Lineage Specification → Redirect Lineage Specification (Aldea et al., 2021; Lu et al., 2016). Pathogenesis: Progenitor cells default to HF lineage due to reduced EN1 activity; ESG density is decreased. Regenerative Strategy: BMP4/5+FGF18+SHH inhibition; CRISPRa-*EN1*; AAV-*EN1.* Tier 3: Aborted Morphogenesis → Ensure Morphogenesis and Maturation (Branch and Ezhkova, 2024; Dingwall et al., 2024). Pathogenesis: ESG lineage correctly specified but gland fails to mature due to EDEN deficiency/absence; immature/dysplastic glands. The molecular cue by which EN1 induces EDEN specification has not been resolved. Regenerative Strategy: EDEN-like fibroblast reprogramming; iPSC-derived niche; synthetic scaffolds. Tier 4: Compromised Adult Structural Integrity → Reinforce Mechanical Anchor (Mainguy et al., 1999; Mainguy et al., 2000). Pathogenesis: Candidate EN1–BPAG1e regulatory relationship supported by *in vitro* data (using EN2 as a surrogate); direct EN1 occupancy and *in vivo* function are untested. Regenerative Strategy: AAV-*BPAG1e*; CRISPRa-*BPAG1e*; EN1 stabilizers/peptides. Colors denote evidence level for pathogenesis: green = experimentally validated in animal models; orange = proposed concept requiring further validation. All regenerative strategies depicted are conceptual and are shown in gray. Abbreviations: EN1, Engrailed-1; ESG, eccrine sweat gland; EDEN, EN1-dependent eccrine niche; HF, hair follicle; SHH, Sonic Hedgehog; BMP, bone morphogenetic protein; FGF, fibroblast growth factor; CRISPRa, CRISPR activation; AAV, adeno-associated virus; iPSC, induced pluripotent stem cell.

First, the four tiers are organized according to the developmental stage at which the EN1 regulatory network is disrupted. Tiers 2 and 3 lie at the core of the ESG developmental program: Tier 2 encompasses failures in lineage specification (the decision to commit to an ESG fate), while Tier 3 encompasses failures in morphogenesis (the subsequent execution of the glandular differentiation program). By contrast, Tier 1 affects a prerequisite developmental event—the establishment of the ventral limb field itself—which occurs before ESG development can even begin. Tier 4, on the other hand, is a post-developmental defect: it occurs after morphogenesis is complete and affects the maintenance of adult gland integrity rather than its formation. These are biologically distinct disease categories with fundamentally different therapeutic logics. Second, the therapeutic window differs fundamentally between congenital and acquired conditions. For congenital hypohidrosis (Tiers 2–3), intervention would ideally target embryonic or early postnatal progenitors that retain competency. For adult burn patients (Tier 1) or acquired ductal damage (Tier 4), regeneration demands either the *de novo* creation of competent progenitors or the reinforcement of existing structures—neither of which can simply recapitulate embryonic programs. Third, the capacity of adult epidermal stem cells to reactivate EN1-dependent developmental programs remains largely unknown, and murine developmental timelines may not translate to human adult tissues. These caveats are integrated into the strategy discussions for each tier. In essence, Tiers 1-3 represent a developmental continuum—from field establishment to lineage choice to morphogenesis—whereas Tier 4 is a post-developmental maintenance defect.

Biomarker candidates: loss of ventral markers (e.g., *EN1*, *BMP7*) and ectopic expression of dorsal markers (e.g., *WNT7A*, *LMX1B*) in affected skin.

### 4.1 Regenerative Strategy

Currently, no established strategy exists for *de novo* establishment of the ventral permissive field in postnatal or adult skin. The embryonic ventral specification program, orchestrated by EN1-mediated repression of *WNT7A*, represents a unidirectional developmental event that has not been shown to be reactivatable in differentiated adult tissues. Achieving spatially restricted reprogramming of dorsal or non-committed skin to a ventral, glabrous identity without affecting adjacent structures poses an even greater challenge. For adult burn patients or patients with congenital ventral field defects, this tier therefore points not to *in situ* reprogramming, but to the development of bioengineered skin substitutes that inherently carry ventral identity—a direction that remains in its early stages but offers the most conceptually coherent path forward.

The evidence underlying these tiers is derived predominantly from mouse studies, with limited human validation (summarized in Table 3). From a diagnostic and genetic perspective, each tier can be conceptually linked to distinct human conditions. Tier 1, though rarely observed as homozygous lethal *EN1* mutations, may be recapitulated by severe congenital limb malformations affecting the ventral ectoderm (e.g., ectrodactyly or syndactyly syndromes with dorsoventral patterning defects), and most commonly by extensive deep burns that destroy volar skin (see Section 4.1). Tier 2 corresponds to congenital hypohidrosis that may be caused by hypomorphic *EN1* alleles, mutations in its activators (*DEAF1*), or enhancer lesions (*ECE18*) based on mouse models and human cell-based assays; however, it should be emphasized that pathogenic variants in *EN1*, *DEAF1*, or *ECE18* have not yet been reported as confirmed causes of human congenital hypohidrosis, and these assignments remain inferential until validated by human clinical genetic evidence. Tier 3 captures rare cases with normal *EN1* expression but aberrant mesenchymal microenvironments or downstream morphogenetic pathways (e.g., *SHH* or *PTCH1* mutations), with EDEN ablation serving as the representative example for “mesenchymal-support-deficient” hypohidrosis. Tier 4, while not a hypohidrotic condition *per se*, manifests as skin fragility disorders (e.g., epidermolysis bullosa simplex) due to *BPAG1e* disruption, which may coexist with mild sweat duct shearing.

### 4.2 Tier 1 — loss of the permissive field (limb patterning defects)

#### 4.2.1 Pathogenesis

This represents a failure at the most fundamental level: the establishment of the ventral, glabrous skin field itself. The hallmark is a complete absence of ESGs alongside severe anatomical changes, such as the transformation of palm/sole skin into a hair-bearing, dorsal-type epidermis, as seen in *En1* knockouts (Loomis et al., 1996). In clinical practice, this tier is most commonly encountered not as a congenital EN1 deficiency, but as an acquired condition—extensive deep burns that physically destroy volar skin, thereby eliminating the ventral permissive field and its resident ESG progenitors (Sheng et al., 2009; Sommer et al., 1998). The developmental logic of Tier 1 thus applies to both congenital field defects and acquired field destruction.

Biomarker candidates: loss of ventral markers (e.g., *EN1*, *BMP7*) and ectopic expression of dorsal markers (e.g., *WNT7A*, *LMX1B*) in affected skin.

#### 4.2.2 Regenerative Strategy

Currently, no established strategy exists for *de novo* establishment of the ventral permissive field in postnatal or adult skin. The embryonic ventral specification program, orchestrated by EN1-mediated repression of *WNT7A*, represents a unidirectional developmental event that has not been shown to be reactivatable in differentiated adult tissues. Achieving spatially restricted reprogramming of dorsal or non-committed skin to a ventral, glabrous identity without affecting adjacent structures poses an even greater challenge. For adult burn patients or patients with congenital ventral field defects, this tier therefore represents the most formidable challenge. Given the unidirectional nature of embryonic ventral specification and the lack of evidence for its reactivation in adult tissue, this tier currently points not to feasible *in situ* reprogramming, but rather to a long-term goal of developing bioengineered skin substitutes that inherently carry ventral identity.

### 4.3 Tier 2 — failure of lineage specification

#### 4.3.1 Pathogenesis

In this scenario, the limb field is anatomically normal, but the specific molecular instruction to commit to the ESG fate is absent or inadequate. The dose-dependent nature of EN1 (see Section 2.1) means that even a partial reduction in *EN1* activity can tip the balance from ESG to HF (Kamberov et al., 2015). However, it should be noted that EN1 heterozygosity may affect additional uncharacterized targets, and the observed phenotype may not be solely attributable to the ESG-HF balance.

Biomarker candidates: preserved glabrous skin architecture with reduced ESG density; decreased *EN1* or its downstream targets (e.g., *DKK4*) in patient skin biopsies.

#### 4.3.2 Regenerative Strategy

The therapeutic goal is to redirect the fate of competent progenitor cells. This could be achieved, in principle, through pharmacological intervention (localized delivery of BMP4/5 and FGF18 combined with SHH inhibition to activate the endogenous *EN1* locus) or gene therapy (CRISPR activation targeting enhancers like *ECE18*, or adeno-associated virus (AAV)-mediated delivery of wild-type *EN1* cDNA under a skin-specific promoter) (Matharu et al., 2019; Wang et al., 2020). However, it is important to emphasize that these strategies have not been validated in ESG-specific models and remain conceptual at this stage. A critical bottleneck for adult burn patients is that the competent embryonic progenitors targeted by these strategies are either absent or refractory to developmental signals in postnatal skin. Unlike the fetal epidermis, where EN1 activation occurs in a permissive chromatin landscape, adult keratinocytes exhibit epigenetic silencing of key developmental loci, and the fibrotic, inflammatory wound milieu actively suppresses morphogenetic programs (Mascharak et al., 2021; Rinkevich et al., 2014). Thus, for Tier 2 interventions to succeed in adults, they must be preceded by an obligate step of epigenetic reprogramming or dedifferentiation of adult epidermal cells to a competent progenitor state—a challenge that remains largely unmet and represents a critical frontier for future investigation. Critically, these approaches remain entirely conceptual for ESG regeneration and have not been validated in any sweat-gland-specific *in vivo* models.

### 4.4 Tier 3 — impaired morphogenesis despite correct specification

#### 4.4.1 Pathogenesis

Here, the ESG lineage is correctly specified, but the gland fails to mature. This tier is exemplified by the failure to induce or maintain the EDEN niche. Genetic ablation of the EDEN in mice shows that while *En1* is expressed, gland morphogenesis is severely aborted (Dingwall et al., 2024). Other examples include failures in later-stage pathways, such as *Shh*-driven coiling or *Foxa1*-mediated functional differentiation (Cui et al., 2012; Cui et al., 2014).

Biomarker candidates: decreased EDEN markers (*S100A4*, *TNC*) in the dermis of affected skin; aberrant expression of morphogenesis regulators (*SHH*, *FOXA1*) in glandular epithelium; normal *EN1* expression in epidermal buds with abortive glandular downgrowth on histology.

Regenerative Strategy: The focus shifts from fate instruction to providing morphogenetic support. Conceptually, this would involve engineering a functional EDEN-like niche to substitute for the transient embryonic stroma. Feasible approaches under investigation include transient reprogramming of resident dermal fibroblasts into an EDEN-like state using niche-signaling factors (e.g., Wnts, TGF-β, BMPs), transplantation of induced pluripotent stem cell (iPSC)-derived EDEN-specified fibroblasts alongside epithelial progenitors, or implantation of biomaterial scaffolds functionalized with key EDEN signals (e.g., BMP5, FGF18, Tenascin-C) (Dingwall et al., 2024). However, these approaches remain experimental and have not been validated in ESG-specific models. The precise molecular nature of the EN1-derived cue that initiates EDEN specification remains unresolved (Section 3.4), and the EDEN is a developmentally restricted transient embryonic structure. Sustained delivery of niche signals beyond the morphogenetic window may be ineffective or even detrimental; spatiotemporally controlled release systems are therefore preferred, but all approaches remain conceptual at this stage.

### 4.5 Tier 4 — compromised adult structural integrity (loss of mechanical support)

#### 4.5.1 Pathogenesis

Unlike the preceding developmental tiers, Tier 4 represents a post-morphogenetic failure of adult gland integrity. EN1 expression is maintained in adult volar keratinocytes, including those of the eccrine ducts, where Engrailed proteins (using EN2 *in vitro*) has been shown to regulate the *Bpag1e* promoter in cultured keratinocytes (Mainguy et al., 1999; Mainguy et al., 2000). Based on the role of BPAG1e in hemidesmosomal adhesion, it has been hypothesized that loss or reduction of this EN1–BPAG1e axis could weaken the dermal-epidermal junction, potentially leading to skin fragility (Guo et al., 1995). However, this hypothesis has not been tested in the context of EN1 regulation of BPAG1e in adult skin.

By analogy, it has been hypothesized that similar mechanical vulnerability at the intraepidermal sweat duct could predispose it to shearing and collapse. Consequently, sweat secretion itself may remain intact, but compromised ductal patency could potentially lead to secondary hypohidrosis and, in severe cases, miliaria-like inflammatory occlusion (Wenzel and Horn, 1998). It is important to note, however, that the ductal collapse model remains entirely speculative—no direct experimental evidence has yet demonstrated that BPAG1e deficiency leads to ductal patency failure *in vivo* or in human tissues (see Table 4). Beyond its structural role, loss of the neural isoform BPAG1n has been associated with degeneration of sympathetic neurons and reduced autonomic nerve fiber density around sweat glands in *dt/dt* mice (Tseng et al., 2011), as elaborated in Section 3.5, although this represents an isoform-distinct neuronal phenotype rather than a defect in epithelial BPAG1e.

#### 4.5.2 Biomarker candidates

Diminished BPAG1e immunofluorescence in glabrous skin biopsies; ultrastructural hemidesmosomal splitting by electron microscopy; normal expression of secretory markers (FOXA1, NKCC1) contrasting with ductal collapse on sweat function tests.

#### 4.5.3 Regenerative Strategy

Since the epithelial lineage and glandular architecture are intact, the goal is to reinforce the mechanical anchor. Feasible approaches that remain conceptual at this stage include: (1) localized small-molecule or peptide therapies that stabilize EN1 protein or enhance its transcriptional activity at the *BPAG1e* promoter; (2) AAV-mediated *BPAG1e* gene supplementation under a keratinocyte-specific promoter for monogenic deficiencies; and (3) CRISPR activation (CRISPRa) targeting the proximal *BPAG1e* regulatory region to bypass dysfunctional EN1-binding motifs, thereby restoring physiologic BPAG1e levels without constitutive EN1 overexpression. Major challenges include achieving durable engraftment of corrected keratinocyte stem cells within the sweat duct niche and avoiding mechanical stress gradients at the borders of mosaic correction, which may paradoxically increase blister risk—issues that must be addressed in preclinical models before clinical application. None of these approaches have been validated in ESG-specific models.

## 5 Limitations, unresolved questions, and translational hurdles

Despite the conceptual clarity of the EN1-centric framework presented here, several critical gaps must be acknowledged.

First, nearly all mechanistic evidence for EN1 function in ESG development comes from mouse models, which differ substantially from human biology. Murine ESGs are restricted to footpads, whereas human ESGs are distributed across the entire body surface; this anatomical difference implies that EN1’s regulatory targets and their spatial control may not be fully conserved. Moreover, the human-specific enhancer ECE18 is absent from the mouse genome, limiting the ability of standard mouse models to fully recapitulate human ESG density regulation. As discussed in Section 4 and summarized in Table 3, the extent to which findings from mouse footpad ESGs translate to human body-wide ESGs remains an open question.

Second, the direct EN1 cistrome in human ESG progenitors has not been defined. While transcriptomic analyses in mice have identified candidate EN1-dependent genes such as *Dkk4* and *Bmp7*, it remains unknown whether EN1 binds directly to their regulatory regions, whether these regulatory relationships are conserved in human progenitors, or whether human-specific targets exist. Defining the EN1 cistrome and its downstream transcriptional network in human fetal glabrous skin through single-cell multi-omics (scRNA-seq + scATAC-seq) and, where possible, tissue-compatible chromatin profiling, is therefore a priority to bridge this gap.

Third, the EDEN model, while elegant and supported by mouse lineage-tracing and ablation experiments, has not been validated in human tissues. Whether a comparable transient dermal niche exists during human ESG development, and whether it is induced by the same EN1-dependent cues, remains to be determined. Similarly, the proposed non-cell-autonomous role of secreted EN1 in EDEN induction remains speculative without conditional alleles that separate EN1’s transcriptional from its paracrine functions.

Fourth, even when human validation is achieved, adult tissue competency presents a fundamental barrier to regenerative translation. The embryonic progenitors that respond to EN1-mediated fate specification are either absent or epigenetically silenced in postnatal skin. The fibrotic and inflammatory milieu of burn wounds actively suppresses morphogenetic programs (Mascharak et al., 2021), and the capacity of adult epidermal stem cells to reactivate EN1-dependent developmental cascades is largely unknown. These challenges are not merely technical but reflect fundamental biological differences between development and adult repair.

Fifth, safety and microenvironmental considerations for EN1-based therapies are substantial. Sustained EN1 overexpression carries oncogenic risk, as EN1 has been implicated in tumorigenesis across multiple cancer types, including basal-like breast cancer (Beltran et al., 2014), triple-negative breast cancer (Peluffo et al., 2019), glioma (Chang et al., 2022), and esophageal squamous cell carcinoma (Zhao et al., 2024). Viral vector delivery may provoke immune responses, and off-target activation of EN1 in non-glabrous skin could induce ectopic gland formation with unpredictable functional consequences. The temporal precision of EN1 activity is also critical: developmental programs operate within narrow temporal windows, and sustained or mistimed activation could produce dysplastic structures rather than functional glands. Beyond these cell-autonomous risks, the role of the tissue microenvironment remains underexplored. Although immune cells have been shown to interact with ESGs in adult tissues—for example, through dendritic cell-mediated metabolic support of secretory cells (Ye et al., 2026) and cytotoxic T lymphocyte infiltration in inflammatory anhidrosis (Sano et al., 2023)—whether immune-mediated signaling plays any role in EN1-dependent ESG development or regeneration remains entirely unknown. Together, these findings underscore the importance of rigorous safety assessment—including long-term tumorigenicity monitoring and evaluation of microenvironmental influences—for any EN1-based therapeutic strategy.

Finally, regulatory and manufacturing barriers to clinical translation remain formidable. Even if proof-of-concept is achieved in animal models, the development of safe, scalable, and cost-effective therapies for ESG regeneration will require substantial investment and interdisciplinary collaboration. These limitations do not diminish the promise of the EN1 framework but underscore the need for rigorous, stepwise translation.

## 6 Conclusion and future perspectives

The evidence consolidated here supports the view that EN1 functions as a central regulator that coordinates ESG development across multiple biological scales—from field establishment and lineage specification to niche induction and adult maintenance. This unified framework offers a conceptual, tier-based roadmap for regenerative therapy.

However, translating this roadmap into clinical reality requires addressing four critical gaps. First, the human EN1 cistrome and interactome in ESG progenitors must be defined via single-cell multi-omics (scRNA-seq + scATAC-seq) on human fetal glabrous skin, as mouse-centric models may miss human-specific targets. Spatial transcriptomics offers the additional advantage of preserving tissue architecture, allowing mapping of EN1 expression gradients relative to the EDEN and surrounding dermal fibroblasts. This technology could resolve whether EN1 expression levels correlate spatially with ESG density and whether EDEN fibroblasts exhibit distinct spatial organization patterns. Second, the relative contribution of cell-autonomous (nuclear) versus non-cell-autonomous (secreted) EN1 to EDEN induction requires lineage-specific conditional knockouts. Third, adult ESG regeneration must achieve not only morphological reconstitution but also functional integration—including cholinergic reinnervation and ductal patency to the skin surface (Ouyang et al., 2018; Zhang L. et al., 2021). Fourth, safety and translational hurdles must be rigorously assessed. Beyond the immunogenicity of viral vectors, the sustained overexpression of EN1 poses a non-negligible oncogenic risk (Beltran et al., 2014; Peluffo et al., 2019). Preclinical studies must therefore include long-term tumorigenicity monitoring and incorporate safety switches or regulatable expression systems.

Emerging technologies offer new avenues to address these challenges. Human skin organoid systems derived from pluripotent stem cells or primary keratinocytes provide a tractable system to model ESG development in a controlled *in vitro* environment. Incorporating EN1 modulation into these systems could test sufficiency and necessity in a human genetic background and serve as a platform for drug screening. Bioengineered skin equivalents with defined dermal and epidermal compartments, incorporating EN1-inducible systems and EDEN-like fibroblast populations, would enable systematic dissection of the epithelial-mesenchymal interactions that govern ESG morphogenesis.

In particular, such models could be designed with spatially patterned EN1 expression to recapitulate the ventral versus dorsal skin field, or with inducible EDEN-like niches to test the sufficiency of specific dermal signals for ESG induction. Advances in 3D bioprinting and decellularized dermal scaffolds offer complementary approaches to generate skin substitutes with integrated, functional eccrine gland structures (Sun et al., 2021; Yao et al., 2020; Zhang Y. et al., 2021). These bioengineered platforms could also serve as preclinical screening tools for the tiered regenerative strategies proposed in this review, providing a controlled human-relevant context for evaluating pharmacological and genetic interventions before animal testing or clinical translation.

Success in this endeavor will not only alleviate the burden of anhidrosis but also illuminate fundamental principles of organogenesis and regeneration applicable beyond the skin. Indeed, the EN1 paradigm—a single transcription factor coordinating field establishment, lineage switching, niche induction, and adult maintenance—may offer a conceptual framework for understanding and potentially guiding the regeneration of other complex ectodermal appendages, including teeth, mammary glands, and HFs.
